# Improving precision instrument cleaning with a quality control module: implementation and outcomes

**DOI:** 10.3389/fpubh.2025.1553113

**Published:** 2025-05-14

**Authors:** Juan Xiong, Wei Jiang, Wei Wan, Li Zuo, Ping Tu, Dong Xia Rao

**Affiliations:** ^1^Department of Emergency, the Second Affiliated Hospital, Jiangxi Medical College, Nanchang University, Nanchang, Jiangxi Province, China; ^2^School of Nursing, Jiangxi Medical College, Nanchang University, Nanchang, Jiangxi Province, China; ^3^Department of Central Sterile Supply Department, the Second Affiliated Hospital, Jiangxi Medical College, Nanchang University, Nanchang, Jiangxi Province, China; ^4^Department of Post Anesthesia Care Unit, the Second Affiliated Hospital, Jiangxi Medical College, Nanchang University, Nanchang, Jiangxi Province, China

**Keywords:** disinfection, central supply, hospital, quality control, artificial intelligence, research

## Abstract

**Objective:**

To evaluate the effectiveness of a cleaning quality control module integrated into a hospital’s Disinfection Supply Center (DSC) for improving the cleaning quality of precision surgical instruments.

**Methods:**

In June 2023, a cleaning quality control module was implemented in our hospital’s DSC to optimize reprocessing procedures. This module recorded deviations through textual, photographic, and video records. A comparative cohort study was conducted on 3,000 instrument sets processed before (February to May 2023) and after (June to September 2023) the intervention. Additionally, 15 DCS technicians were assessed for theoretical understanding and practical proficiency. Statistical analyses compared cleaning success rates, clinical user satisfaction, and technicians’ performance.

**Results:**

Cleaning qualification rates for instrument surfaces, accessories, and lumens increased from 97.5, 96.9, and 95.8% to 99.9, 99.2, and 98.2%, respectively. ATP bioluminescence results also improved, with rates rising from 97.1, 96.2, and 94.9% to 99.1, 98.7, and 97.9%. Clinical technicians’ satisfaction rose from 91 to 99%. Technicians theoretical and operational scores improved from 89.20 ± 3.35 to 97.10 ± 1.55 and from 90.10 ± 4.33 to 97.50 ± 1.00, respectively. All differences were statistically significant (*p* < 0.05).

**Conclusion:**

The cleaning quality control module significantly enhanced instrument cleaning quality, improved technicians training efficiency, enhanced departmental operational efficiency, extended instrument lifespan, and improved patient safety.

## 1 Introduction

The hospital’s Disinfection Supply Center (DSC) is responsible for cleaning, disinfecting, sterilizing, and supplying sterile items for reusable diagnostic and treatment instruments across various departments (1, 2). The growing use of advanced medical equipment, due to advances in precision medicine and minimally invasive surgery, has increased challenges in handling precision instruments (3–5). These instruments are diverse, intricately designed, fragile, valuable, limited in availability, and have high turnover rate, further complicating reprocessing procedures (2, 6). Ensuring reprocessing quality has thus become critical, requiring stringent quality control and monitoring (4).

Globally, hospitals are adopting advanced information systems for end-to-end monitoring and quality tracking of precision instruments (7–10). For instance, in the United States, the implementation of Sterile Processing Department (SPD) tracking systems has significantly improved the traceability and quality control of surgical instruments (7). These systems integrate barcode scanning and real-time data logging to ensure compliance with standards such as AAMI ST79 and ISO 17664 (8, 9). Similarly, in China, hospitals have adopted DSC management systems that utilize RFID technology for instrument tracking and automated cleaning validation, enhancing both efficiency and patient safety (10). These global efforts highlight the importance of advanced information systems in improving the quality and safety of precision instrument cleaning, providing valuable insights for the implementation of similar systems in other healthcare settings.

However, despite these advancements, many hospitals in China still face significant challenges in achieving comprehensive digitalization. Most hospitals use limited digitalization modules that record data only at specific workflow nodes. Incompatibilities with cleaning and sterilization equipment or high interface costs mean many centers rely on manual documentation, leading to issues like lost records, inefficient data retrieval, incomplete information, insufficient data validation, and poor cost control (5, 6). Additionally, these modules often lack advanced features like rapid batch recall and automated alerts, and operate independently of HIS (11), hindering end-to-end quality tracking, especially during healthcare-associated infections.

To address these issues, in June 2023, our hospital’s DSC, in collaboration with the information department and manufacturer engineers, added a cleaning quality control module to the existing quality tracing system. Its application significantly improved precision instrument cleaning quality, with the results detailed in the following section.

## 2 Materials and methods

### 2.1 General information

#### 2.1.1 Instrument data

Between February and May 2023 (pre-implementation) and June to September 2023 (post-implementation), 1,500 sterile instrument sets were randomly selected from each period for statistical evaluation in the DSC. The 1,500 packs processed before module application were assigned to the control group, while those processed after module application were designated as the intervention group. There were no statistically significant differences between the two groups in terms of frequency of use, frequency of cleaning, or other baseline characteristics (*p* > 0.05) (12).

#### 2.1.2 Personnel data

Fifteen DSC technicians who involved in instrument processing were selected and evaluated for both theoretical understanding and practical proficiency over two distinct time periods. The age range of the participants was from 38 to 50 years, with a mean age of 46.7 years (SD = 5.26). The sample size was determined in accordance with statistical principles, with a confidence level of 95% and a statistical power of 80%, thereby ensuring the reliability of the study results. Stratified sampling was employed to select the sample, ensuring representativeness of the entire DSC technician population in terms of key characteristics, including age, gender, and work experience.

### 2.2 Research method

#### 2.2.1 Compliance with ISO 17664

This study adhered to the guidelines outlined in ISO 17664:2021, which provides a framework for the development and validation of cleaning and disinfection protocols for medical devices. The standard ensures that the cleaning process is effective, reproducible, and well-documented.

**Documentation of Cleaning Procedures**: Detailed cleaning protocols were developed, including pre-cleaning, main cleaning, rinsing, and drying steps, as recommended by ISO 17664.

**Validation of Cleaning Efficacy**: The cleaning process was validated using [specific methods, e.g., microbial testing, ATP bioluminescence], ensuring compliance with the acceptance criteria outlined in ISO 17664 (e.g., microbial load reduction by X log, protein residue < Y μg/cm^2^).

**Risk Management**: Potential risks associated with the cleaning process (e.g., residual contaminants, cross-contamination) were identified and mitigated through the implementation of real-time monitoring and quality control measures.

#### 2.2.2 Module development and team formation

The team was comprised the head nurse of the DSC, the quality control nurse of the DSC, the traceability module software engineer, the IT department engineer, and several head nurses from clinical departments. The head nurse of the DSC defined the functional requirements for the module and coordinated related work. The traceability module software engineer was responsible for software design, maintenance, and training relevant personnel on the use of the module. The IT department engineer was responsible for the integration and maintenance of the software with the hospital’s module. The quality control nurse of the DSC oversaw the implementation process and gathered data including the qualification rate of precision instrument cleaning, satisfaction of clinical department technicians, and theoretical and operational scores of the technicians, after all personnel received uniform training on data collection methods. The head nurses of various clinical departments monitored instrument usage in their respective departments and ensured that nursing staff could correctly upload issues and images through the module’s clinical feedback feature, which was easily mastered after initial training.

#### 2.2.3 Construction of the module

**Figure 1:**
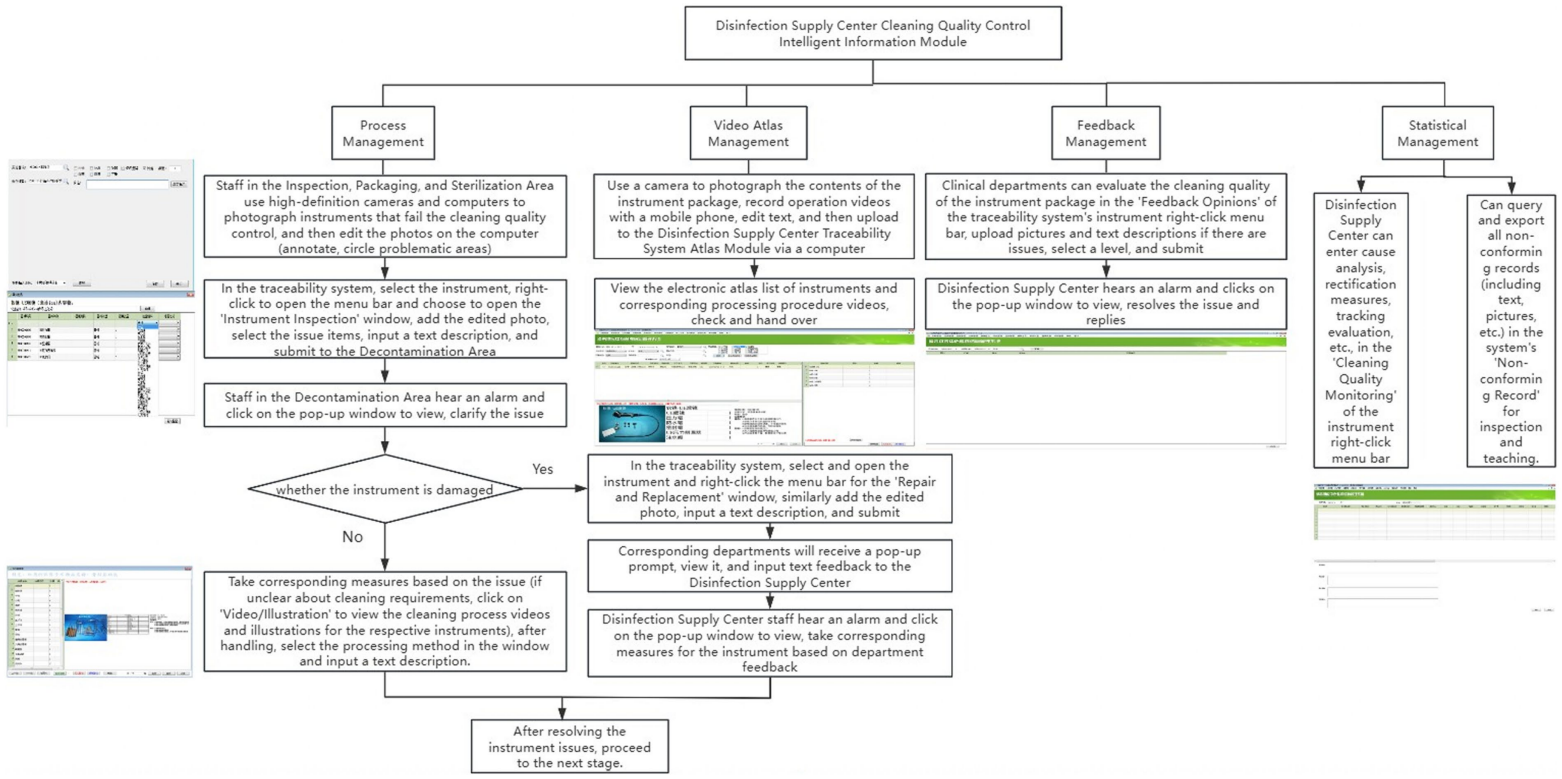
Workflow diagram of the disinfection supply center cleaning quality control intelligent information module.

To overcome existing limitations, a customized version of the Xinhua Sterilization Quality Tracing module, developed by Shandong Xinhua Medical Instrument Co., Ltd. in 2009, was incorporated into the existing traceability infrastructure of the Disinfection Supply Center in 2020. The existing system comprised modules for login, basic data management, recovery management, cleaning management, assembly management, sterilization management, distribution management, warehouse management, and equipment monitoring. The new cleaning quality control module was added to enhance these capabilities, its key functions are summarized in Table 1. Figure 1 illustrates the key functions of the new cleaning quality control module, illustrating how it integrates with the existing system and improves the overall traceability of quality control processes.

**Table 1:** Key functions of the cleaning quality control module.

Feature no.	Function description
1	**Real-time Monitoring**: Monitor critical cleaning parameters (e.g., time, temperature, pressure) via HIS integration to ensure compliance with ISO 17664 standards (41).
2	**High-Definition Imaging**: Use HD cameras to photograph instruments and allow annotations on the computer.
3	**Cleaning Quality Assessment**: Flag non-conforming instruments and record issues with text and images.
4	**Non-Conforming Instrument Alerts**: Trigger red warnings and alarms for non-conforming instruments until resolved.
5	**Problem Handling and Feedback**: View non-conformance reasons, select handling methods, and provide additional feedback.
6	**Process Visualization**: Link to video/graphic explanations of cleaning processes via the instrument atlas module.
7	**Specialty Instrument Management**: Transfer instruments needing repair/replacement to relevant departments with photos and explanations.
8	**User Department Feedback**: Allow departments to evaluate instrument packs, upload photos, and select problem levels (Level 1 triggers alerts).
9	**Summary Query for Non-Conforming Cleaning**: Search and sort non-conforming cleaning records by various criteria.
10	**Data Export**: Export all related files and data statistics (reports, images, videos, etc.).

#### 2.2.4 Hardware configuration

Additionally, the decontamination, inspection, packaging, and sterilization zones are equipped with 10 Lenovo Yangtian S4250-00 computers featuring HD cameras, manufactured in November 2017 by Lenovo (Beijing) Co., Ltd. For visual documentation, the center uses a Canon EOS R6 Mark II high-definition digital camera, released in November 2022 by Canon (China) Co., Ltd., and an Apple iPhone 14, released in September 2022 by Apple Inc. In the decontamination area, inspection, packaging, and sterilization area, there are 10 computers equipped with high-definition cameras and 1 high-definition digital camera.

#### 2.2.5 Personnel training

Following the implementation of the cleaning quality control module, software engineers organized centralized training sessions for relevant technicians at the Disinfection Supply Center. The training combined practical demonstrations with theoretical guidance, covering basic operating procedures, photography techniques, image editing methods, and essential precautions. After the training, quality control nurses assessed the trainees, conducted random queries, and identified areas requiring additional training. They ensured that all personnel mastered the module’s operational procedures and could effectively apply them in their daily tasks. For clinical departments, representatives were required to attend centralized online training sessions. Subsequently, they instructed their departmental personnel to ensure proficiency in feedback procedures.

#### 2.2.6 Specific application

The intelligent information module enables real-time tracking of critical parameters (e.g., time, temperature, pressure, detergent concentration) during cleaning operations, ensuring compliance with predefined standards. Non-compliance triggers red pop-up warnings and alarms, prompting staff to investigate and resolve issues before reprocessing.

In inspection, packaging, and sterilization areas, technicians document non-conforming instruments by capturing and annotating images to highlight defects. These images and descriptions are uploaded to the traceability module, where decontamination personnel review alerts, implement corrective measures, and access instructional resources if needed. Instruments requiring repair or replacement are flagged in the system.

Clinical departments can submit feedback on cleaning quality through the module, including photos, descriptions, and ratings. The Disinfection Supply Center reviews feedback, implements corrective actions, and documents analysis and evaluations. All non-conforming records are stored for inspection and training.

### 2.3 Evaluation indicators

#### 2.3.1 Cleaning qualification rate

1. Visual Inspection: The cleaning quality of the surfaces, lumens, and accessories of precision instruments is visually inspected or with a magnifying glass. Qualification is assessed based on cleanliness, brightness, dryness, and absence of watermarks, lack of stains or blood residue, no rust, unobstructed lumens, and good appearance; failure in any aspect leads to disqualification.
2. ATP Detection Method:
  - Surface Sampling: Use a sampling swab to evenly swab the surface of the instrument and its accessories, then insert the swab into the 3 M Clean-Trace ATP detection device’s test tube and start the detector to conduct the test. The results are read and recorded after they appear.
  - Lumen Sampling: Align one end of the instrument lumen with the sample collection cup (carefully avoiding contact with the cup or liquid), use a 50 mL syringe to draw 30 mL of sterile water to inject from the other end of the instrument lumen, then inject 40 mL of air after the injection (13), collecting the liquid thoroughly; then use a sampling swab for sampling, insert into the 3 M Clean-Trace ATP detection device’s test tube, and initiate the detector to perform the test. The results are read and recorded after they appear.

The standard for both methods is: values ≤ 20 RLU/strip are qualified (14); otherwise, they are non-qualified.

#### 2.3.2 Instrument rewashing rate

Precision instruments displaying any of the following condition—rust stains, contaminants, incomplete drying, blood residues, or obstructed lumens—when observed directly or with a magnifying glass, must undergo rewashing.

#### 2.3.3 Instrument handover error rate

Issues with precision instruments, including quantity discrepancies (missing or extra items, or missing parts), incorrect disassembly, or mismatches between instruments and their labels, are classified as handover errors.

#### 2.3.4 Instrument wear and tear rate

Issues including detached coatings, misaligned or bent sharp parts, fractured tips, or blunt blades identified during inspection are categorized as wear and tear in precision instruments.

#### 2.3.5 Instrument handover and manual cleaning time

The handover duration (from receiving instruments to completing counting and classification) and manual cleaning duration (including rinsing, washing, soaking, final rinse, and drying) per precision instrument package must be measured and documented.

#### 2.3.6 Sterilization qualification rate

The sterilization status of instruments is evaluated using the throat swab culture method. Swabs soaked in sterile saline are applied to areas difficult to clean and sterilize, such as joints, serrations, and inner lumen walls. After a 72-h incubation period, the presence or absence of colony growth determines the sterilization outcome: no growth indicates successful sterilization, whereas any growth indicates sterilization failure.

#### 2.3.7 Satisfaction of clinical department personnel

To evaluate the satisfaction of clinical medical technicians with instrument usage, an anonymous electronic survey was conducted. The survey included required questions with five response choices: very satisfied, satisfied, neutral, dissatisfied, and very dissatisfied (15). A dedicated WeChat group was established to explain the survey’s purpose and completion requirements. Respondents accessed the questionnaire via a QR code and were required to complete all items before submission. Non-respondents were followed up to ensure participation (16).

Before the implementation of the module, the survey was conducted until 100 valid responses were obtained. After module implementation, a follow-up survey was conducted among the same 100 respondents, and data were analyzed. The satisfaction rate was calculated using the formula: (number of neutral + satisfied + very satisfied responses) / total number of questionnaires × 100% (17).

#### 2.3.8 Theoretical and operational scores of technicians

1. Theoretical Evaluation: Theoretical knowledge on precision instrument cleaning was converted into questions, input into the Questionnaire Star module to create a question bank, and a standardized test paper was created. Relevant technicians were evaluated onsite and scores were compiled, with the scoring module set as a percentage scale (18).
2. Practical Evaluation: According to the precision instrument cleaning operation scoring standard assessment table, relevant technicians were assessed onsite and scores were recorded, using a scoring system based on a percentage scale.

#### 2.3.9 Retraining rate

Participants who score 95 or above in both theoretical and practical evaluations after the initial training are exempted from retraining. Those who score below 95 must participate in additional training sessions on a weekly basis until they meet the required standard. The number of participants and the frequency of retraining sessions should be recorded to calculate the retraining rate.

### 2.4 Statistical methods

Statistical analysis of the data was conducted using IBM SPSS Statistics 26.0 software, released by the International Business Machines Corporation in April 2019. Count data were expressed by frequency and percentage, and inter-group comparisons were performed using the χ^2^ test; measurement data were expressed by mean ± standard deviation, and inter-group comparisons were made using the *t*-test. *p* < 0.05 was considered that the difference was statistically significant.

## 3 Results

### 3.1 Comparison of cleaning qualification rates for precision instruments in both groups

The cleaning qualification rates of precision instrument surfaces, accessories, and lumens in the control group, assessed by visual inspection, were 97.5, 96.9, and 95.8%, respectively. In the intervention group, these rates were 99.9, 99.2, and 98.2%, respectively. Using the ATP bioluminescence method, the cleaning qualification rates of the control group for precision instrument surfaces, accessories, and lumens were 97.1, 96.2, and 94.9% respectively; in the intervention group, these rates were 99.1, 98.7, and 97.9%. All differences were statistically significant (all *p* < 0.05), as shown in Table 2.

**Table 2:** Comparison of reusable precision instrument cleaning pass rates before and after application of the cleaning quality control module [n (%)].

Group	Visual inspection			ATP bioluminescence assay		
	Surface compliance	Accessory compliance	Lumen compliance	Surface compliance	Accessory compliance	Lumen compliance
Control group	1,463(97.5)	1,454(96.9)	1,437(95.8)	1,456(97.1)	1,443(96.2)	1,424(94.9)
Intervention group	1,499(99.9)	1,488(99.2)	1,473(98.2)	1,486(99.1)	1,480(98.7)	1,468(97.9)
χ^2^	34.543	20.324	14.845	15.823	18.248	18.595
*p*	<0.001	<0.001	<0.001	<0.001	<0.001	<0.001

### 3.2 Comparison of precision instrument rewashing rates between groups

The rewashing rate of precision instruments in the control group was 5.5%, while that in the intervention group was 1.2%. This suggests that the rewashing rate in the intervention group was significantly lower than in the control group, and the difference was statistically significant (*p* < 0.05), as shown in Table 3.

**Table 3:** Comparison of the backwash rate of precision instruments between the two groups [pcs (%)].

Group	Number	Rust present	stains present	Not dried	Bloodstains present	Lumens not patent	Total rewash
Control group	1,500	18(1.2)	19(1.3)	37(2.5)	5(0.3)	3(0.2)	82(5.5)
Intervention group	1,500	5(0.3)	2(0.1)	9(0.6)	1(0.1)	1(0.1)	18(1.2)
χ^2^	–	–	–	–	–	–	42.372
*P*	–	–	–	–	–	–	<0.001

### 3.3 Comparison of precision instrument handover error rates between groups

The handover error rate for precision instruments in the control group was 6.9%, compared to 1.4% in the intervention group. The intervention group had a significantly lower handover error rate compared to the control group, and the difference was statistically significant (*p* < 0.05), as detailed in Table 4.

**Table 4:** Comparison of error rates in the handover of precision instruments between two groups [Number (%)].

Group	Number	Discrepancy in quantity	Improper Disassembly	Discrepancy between actual item and identification	Total handover errors
Control group	1,500	63(4.2)	21(1.4)	19(1.3)	103(6.9)
Intervention group	1,500	17(1.1)	4(0.3)	0(0.0)	21(1.4)
χ^2^	–	–	–	–	56.564
*p*	–	–	–	–	<0.001

### 3.4 Comparison of precision instrument wear and tear rates between groups

The wear and tear rate for precision instruments was 2.9% in the control group and 0.5% in the intervention group. The intervention group had a significantly lower wear and tear rate compared to the control group, and the difference was statistically significant (*p* < 0.05), as shown in Table 5.

**Table 5:** Comparison of wear and tear rates of precision instruments between two groups [Number (%)].

Group	Number	Surface coating detachment	Sharp edge misalignment	Sharp edge rolling	Sharp edge fracture	Blade dulling	Total occurrences
Control group	1,500	20(1.3)	12(0.8)	3(0.2)	1(0.1)	7(0.5)	43(2.9)
Intervention group	1,500	5(0.3)	1(0.1)	0(0.0)	0(0.0)	1(0.1)	7(0.5)
χ^2^	–	–	–	–	–	–	26.359
*p*	–	–	–	–	–	–	<0.001

### 3.5 Comparison of handover and manual cleaning times for precision instruments between groups

**Figure 2:**
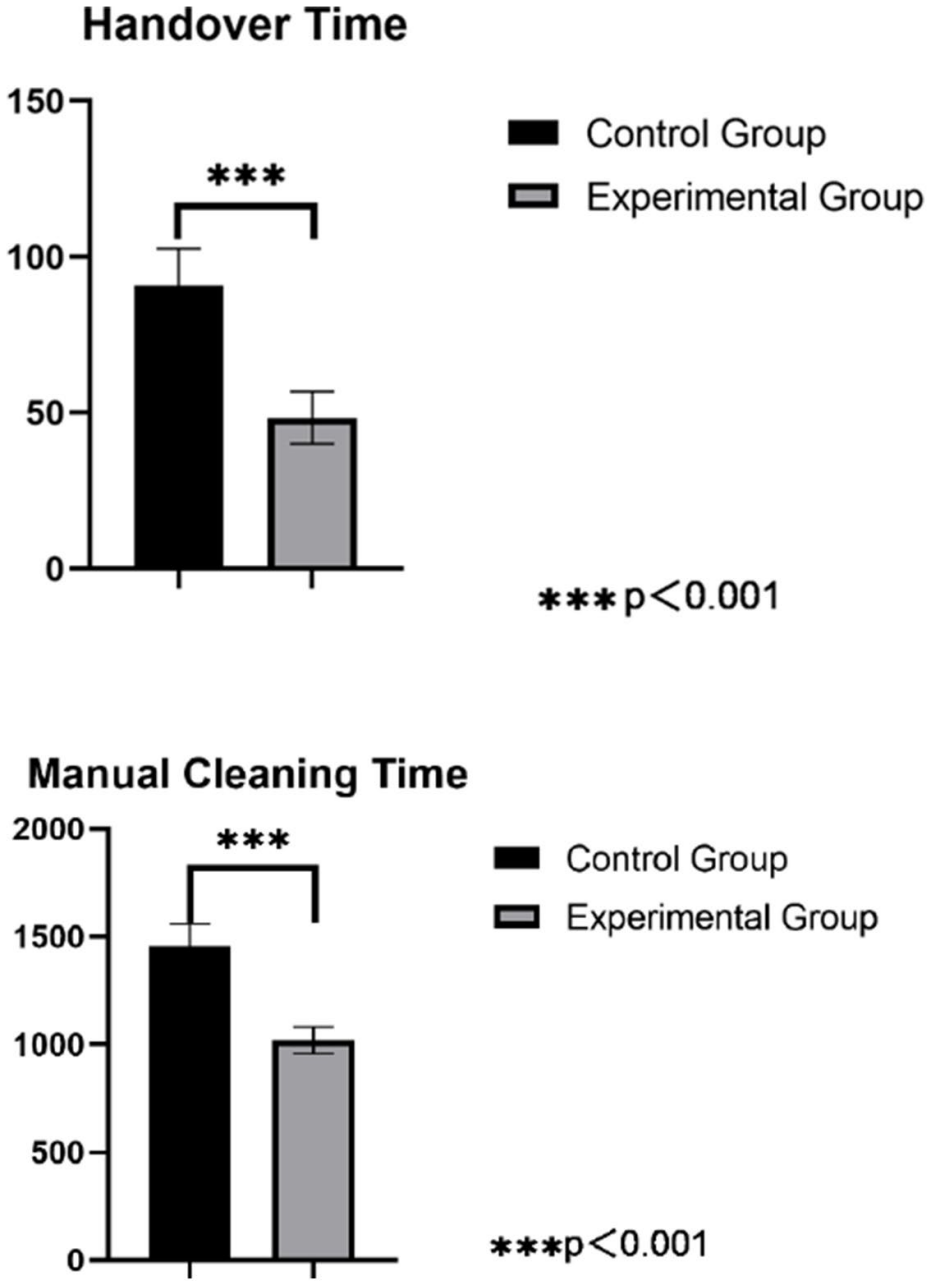
Comparison of handover and manual cleaning times for precision instruments.

In the control group, the handover time for precision instruments was (90.86 ± 11.88) seconds, and the manual cleaning time was (1456.06 ± 102.94) seconds. In the intervention group, the handover time was (48.44 ± 8.43) seconds, and the manual cleaning time was (1021.22 ± 61.85) seconds. The intervention group had significantly shorter handover and manual cleaning times compared to the control group, and both differences were statistically significant (*p* < 0.05), as detailed in Figure 2.

### 3.6 Comparison of sterilization qualification rates for precision instruments between groups

The sterilization qualification rate for precision instruments in the control group was 95.5%, compared to 100.0% in the intervention group. The intervention group had a significantly higher sterilization qualification rate compared to the control group, and the difference was statistically significant (*p* < 0.05), as shown in Table 6.

**Table 6:** Comparison of sterilization qualification rates for two groups of precision instruments [Number (%)].

Group	Number	Sterilization qualified
Control group	1,500	1,432(95.5)
Experimental group	1,500	1,500(100.0)
χ^2^	–	69.577
*p*	–	<0.001

### 3.7 Comparison of satisfaction with cleaning quality of precision instruments among clinical department technicians in both groups

The satisfaction rate of clinical department technicians with the cleaning quality of precision instruments in the control group was 91%. In the intervention group, this satisfaction rate was 99%. The satisfaction rate among clinical department technicians in the intervention group was significantly higher than that in the control group, and the differences were statistically significant (*p* < 0.05), as shown in Table 7.

**Table 7:** Comparison of satisfaction with cleaning quality of precision instruments among clinical department technicians in both groups [units (%)].

Group	Number	Highly satisfied	Satisfied	Neutral	Dissatisfied	Highly dissatisfied	Overall satisfaction rate
Control group	100	15(15.0)	31(31.0)	45(45.0)	8(8.0)	1(1.0)	91(91.0)
Intervention group	100	25(25.0)	34(34.0)	40(40.0)	1(1.0)	0(0.0)	99(99.0)
t	–	–	–	–	–	–	6.737
*p*	–	–	–	–	–	–	0.009

### 3.8 Comparison of theoretical and operational scores of technicians on cleaning of precision instruments in both groups

**Figure 3:**
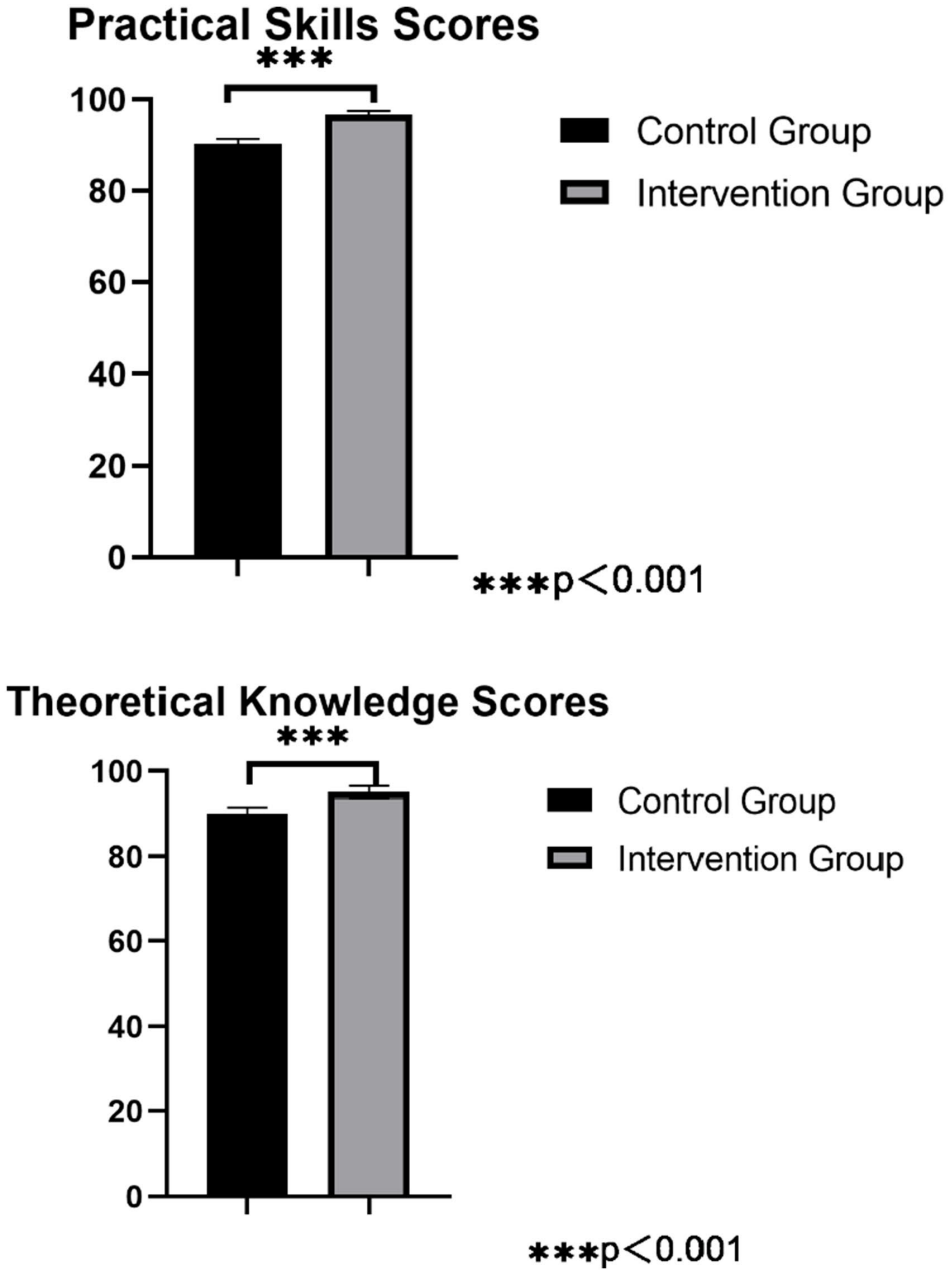
Comparison of practical skills score and theoretical knowledge scores for precision instruments cleaning among workers.

The theoretical scores of the technicians in the control group were 89.20 ± 3.35 points, and the operational scores were 90.10 ± 4.33 points. In the intervention group, the theoretical scores were 97.10 ± 1.55 points, and the operational scores were 97.50 ± 1.00 points. Both the theoretical and operational scores of the technicians in the intervention group were significantly higher than those in the control group, and the differences were statistically significant (*p* < 0.05), as detailed in Figure 3.

### 3.9 Comparison of technicians retraining rates between groups

The retraining rate for technicians in the control group was 100%, compared to 6.7% in the intervention group. The intervention group had a significantly lower retraining rate than the control group, and the difference was statistically significant (*p* < 0.05), as detailed in Table 8.

**Table 8:** Comparison of retraining rates among technicians in two groups [Number (%)].

Group	Number	1 Training session	2 Training sessions	3 Training sessions	Retraining status
Control group	15	0(0.0)	10(66.7)	5(33.3)	15(100.0)
Intervention group	15	14(93.3)	1(6.7)	0(0.0)	1(6.7)
χ^2^	–	–	–	–	26.250
*p*	–	–	–	–	<0.001

## 4 Discussion

Improvements in cleaning and sterilization success rates, reduced reprocessing and handoff error rates, and extended instrument lifespans collectively enhance infection control. By ensuring higher standards of instrument reprocessing, the intervention group showed potential to reduce healthcare-associated infections (HAIs). According to the WHO Guidelines on Core Components of Infection Prevention and Control Programs (19), cleaning and sterilization are fundamental components of infection prevention, and inadequate processes can lead to pathogen transmission and increased HAIs. The significant reduction in rewash rates and handover errors minimize the risk of residual contaminants, which are a known source of HAIs. Additionally, the improved sterilization qualification rate (100% in the intervention group) further mitigates the risk of infection transmission through inadequately sterilized instruments. These findings align with WHO recommendations, which emphasize the importance of standardized cleaning and sterilization protocols, regular monitoring, and feedback mechanisms to ensure effectiveness.

The implementation of the cleaning quality control module not only optimizes operational efficiency but also has broader implications for patient safety and public health. As highlighted by WHO, improving cleaning and sterilization processes can significantly reduce HAIs, lower healthcare costs, and improve patient outcomes. Specifically, the reduction in HAIs can lead to decreased morbidity and mortality rates, contributing to global efforts to combat antimicrobial resistance and enhance public health security. This study underscores the critical role of stringent cleaning protocols in mitigating the spread of infections within healthcare settings, thereby supporting the global IPC goals outlined by WHO.

Quality management methods have gradually evolved from manual entry to information-based quality traceability systems (20). Informational quality traceability modules are being progressively applied in medical institutions at all levels. An informational quality traceability module is an important tool for Disinfection Supply Centers in hospitals to implement process quality management (21). By utilizing barcode and QR code information traceability management technologies, it enables full-process information traceability from instrument recovery to clinical use, achieving closed-loop management of reusable medical instruments. It is also the best means of quality management, improving personnel role management and quality control (22), and providing a scientific basis for the standardized management of hospital Disinfection Supply Centers. Disinfection Supply Centers can use informational means to directly extract basic data of key links such as cleaning, disinfection, packaging, and sterilization. This represents an inevitable trend in quality indicator monitoring (23–25). Therefore, how to develop a comprehensive information-based quality traceability system tailored to the operational realities of Disinfection Supply Centers has become the current focal difficulty. More and more scholars and studies at home and abroad are beginning to focus on how to ensure the reprocessing quality of precision instruments by optimizing the informational quality traceability module of Disinfection Supply Centers (26, 27).

This study added a cleaning quality control module to the existing system, expanding its coverage and improving its functions. The module significantly enhanced the cleaning quality of precision instruments.

1. The Cleaning Quality Control Module Improves the Work Efficiency of the Disinfection Supply Center. The study demonstrates that the implementation of the cleaning quality control module significantly improved the qualification rates for cleaning precision instruments, including their surfaces, accessories, and lumens. Concurrently, the module reduced rewashing rates, handover errors, handover time, and manual cleaning time. This improvement is attributed to the module’s ability to facilitate real-time communication between inspection, packaging, and sterilization technicians, who can now submit issue reports via the traceability module. This system triggers computer alerts with audible alarms, ensuring timely resolution of problems and minimizing delays in instrument turnover (28). These findings are consistent with prior studies (29, 30), which highlight that enhanced traceability modules and visual mapping reduce configuration errors, decrease rewashing frequency, and improve cleaning accuracy and quality. The results also align with the recommendations of William A. Rutala (31), who emphasized that checkpoint alerts and process safeguards are critical for ensuring the quality and safety of reusable sterile items.
2. The Cleaning Quality Control Module Extends the Service Life of Instruments. The results of this study indicate that after implementing the module, the wear and tear rate of precision instruments decreased from 2.9 to 0.5%. The reasons for this are analyzed as follows: after the application of the module, key monitoring of the precision instrument cleaning process and recording of related issues, including text, images, and videos, became possible. By regularly exporting related content, it provides foundational data for identifying key issues, supporting departmental quality control and facilitating continuous improvement, further standardizing the work of related personnel, reducing unnecessary wear and tear on instruments, and extending their service life (24, 26). This finding is consistent with Jing Zhang (32), who demonstrated that an information-based visual monitoring module for traceability effectively controls key failure-sensitive indicators during the handling of reusable instruments.
3. The Cleaning Quality Control Module Increases the Satisfaction of Clinical Department Technicians. The results of this study showed that after the application of the module, the satisfaction level of clinical department technicians with the cleaning quality of precision instruments increased from 91.0 to 99.0%. Another contributing factor is the module’s provision of a communication interface between clinical departments and the sterilization supply center. This feedback mechanism enables timely resolution of instrument-related issues, enhancing collaboration and service quality. These results align with Ting Hu (33), who emphasized the importance of constructive feedback from clinical departments to promote integrated development with sterilization supply centers. The new module provides a communication channel with clinical departments, allowing for feedback on instrument issues between clinical departments and the Disinfection Supply Center and timely handling, promoting more harmonious interactions and improving service quality.
4. The Cleaning Quality Control Module Shortens the Training Time for New Technicians. The results of this study showed that after the application of the module, both the theoretical and operational scores of technicians significantly improved. The retraining rate of technicians has also been significantly reduced. The reasons for this are analyzed as follows: through visual atlas teaching, new nurses can intuitively understand the structure, quantity, shape, function, and precautions of instruments, enhancing memory retention and comprehension; through video-based teaching, new nurses can clearly grasp the key points and difficulties of the disassembly, cleaning, and maintenance of instruments. This reduces the difficulty of expression for the teaching technicians, makes explanations more engaging, improves the quality of teaching, and enables new nurses to quickly familiarize themselves with and master relevant instruments in the shortest time, significantly enhancing their problem-solving skills (34–36). This outcome aligns with previous studies (37, 38), which suggest that visualized mapping and video-based teaching enable new nurses to gain an initial understanding of instruments, facilitating memory retention and comprehension. This approach instructional burden for mentors and significantly accelerates the learning process for new nurses, aiding their transition into their roles. Similarly, the findings are in agreement with Gao Lina (39), who found that optimizing information traceability modules significantly improves the overall competence of sterilization supply center technicians.

## 5 Conclusion

In summary, the application of the cleaning quality control module is worthy of in-depth exploration and promotion. It can improve the cleaning quality of precision instruments, help efficiently train new technicians, further standardize technicians’ work, improve work efficiency, extend the service life of instruments, enhance the satisfaction of clinical department technicians, and ensure patient safety (40). However, this study still has certain limitations, as the current module only includes relevant atlases and videos of precision instruments. The scope can be further expanded to gradually include all instruments, to achieve broader applicability.

## Data Availability

The raw data supporting the conclusions of this article will be made available by the authors, without undue reservation.

## References

[1] Basu D Bhattacharya S Mahajan A Ramanan VR Chandy M. The importance of the central sterile supply department in infection prevention and control. Infect Control Hosp Epidemiol. (2014) 35:1312–4. doi: 10.1086/678072, PMID: 25203193

[2] Chen H Liu J Zhang M. Incidence of adverse events in central sterile supply department: a single-center retrospective study. Risk Manag Healthc Policy. (2023) 16:1611–20. doi: 10.2147/RMHP.S423108, PMID: 37614961 PMC10443689

[3] Malchesky PS Chamberlain VC Scott-Conner C Salis B Wallace C. Reprocessing of reusable medical devices. ASAIO J. (1995) 41:146–51. 7640417

[4] Alfa MJ. Monitoring and improving the effectiveness of cleaning medical and surgical devices. Am J Infect Control. (2013) 41:S56–9. doi: 10.1016/j.ajic.2012.12.006, PMID: 23622750

[5] Santos BF Hungness ES. Natural orifice translumenal endoscopic surgery: progress in humans since white paper. World J Gastroenterol. (2011) 17:1655–65. doi: 10.3748/wjg.v17.i13.1655, PMID: 21483624 PMC3072628

[6] Aronson JK Heneghan C Ferner RE. Medical devices: definition, classification, and regulatory implications. Drug Saf. (2020) 43:83–93. doi: 10.1007/s40264-019-00878-3, PMID: 31845212

[7] Alfred M Catchpole K Huffer E Fredendall L Taaffe KM. Work systems analysis of sterile processing: assembly. BMJ Qual Saf. (2021) 30:271–82. doi: 10.1136/bmjqs-2019-010740, PMID: 33077512 PMC7979531

[8] Bridgman E. AAMI (Association for the Advancement of medical instrumentation) completes recommended practice on decontamination. J Healthc Mater Manage. (1991) 9:78–9. 10108902

[9] Schneider F Maurer C Friedberg RC. International Organization for Standardization (ISO) 15189. Ann Lab Med. (2017) 37:365–70. doi: 10.3343/alm.2017.37.5.365, PMID: 28643484 PMC5500734

[10] Zhu X Yuan L Li T Cheng P. Errors in packaging surgical instruments based on a surgical instrument tracking system: an observational study. BMC Health Serv Res. (2019) 19:176. doi: 10.1186/s12913-019-4007-3, PMID: 30890128 PMC6425664

[11] Zhang XH Xu YL Pan JR Huang BZ Cai LR Wang XX. Current status of the implementation of information-based quality tracing in sterilization supply centers in 129 hospitals. Nurs Res. (2024) 38:2787–91. doi: 10.12102/j.issn.1009-6493.2024.15.026

[12] Hoefel HHK Pozzer C Acunã A Arsego M Bernardo R Castro ME. Bundles for the central sterile supply department. Am J Infect Control. (2019) 47:1352–7. doi: 10.1016/j.ajic.2019.05.010, PMID: 31324496

[13] Kong X Zhu X Zhang Y Wu J. The application of plan, do, check, act (PDCA) quality management in reducing nosocomial infections in endoscopy rooms: it does work. Int J Clin Pract. (2021) 75:e14351. doi: 10.1111/ijcp.14351, PMID: 33973325

[14] Fann LY Cheng CC Chien YC Hsu CW Chien WC Huang YC. Effect of far-infrared radiation on inhibition of colonies on packaging during storage of sterilised surgical instruments. Sci Rep. (2023) 13:8490. doi: 10.1038/s41598-023-35352-9, PMID: 37231027 PMC10212960

[15] Meng S Kong F Dong W Zhang Y Yu T Jin X. Mobile social media use and life satisfaction among adolescents: a moderated mediation model. Front Public Health. (2023) 11:1117745. doi: 10.3389/fpubh.2023.1117745, PMID: 38094229 PMC10716310

[16] Gao Z Ying S Liu J Zhang H Li J Ma C. A cross-sectional study: comparing the attitude and knowledge of medical and non-medical students toward 2019 novel coronavirus. J Infect Public Health. (2020) 13:1419–23. doi: 10.1016/j.jiph.2020.06.031, PMID: 32653479 PMC7328549

[17] Niu Z Huang L He H Mei S Li L Griffiths MD. The revised patient satisfaction questionnaire (PSQ-R): validity, reliability, equivalence, and network analysis among hospitalized patients in the Chinese population. BMC Health Serv Res. (2024) 24:1289. doi: 10.1186/s12913-024-11788-1, PMID: 39468570 PMC11520068

[18] Yang T Lin S Xie Q Ouyang W Tan T Li J. Impact of 3D printing technology on the comprehension of surgical liver anatomy. Surg Endosc. (2019) 33:411–7. doi: 10.1007/s00464-018-6308-8, PMID: 29943060

[19] World Health Organization (WHO). Guidelines on Core components of infection prevention and control Programmes at the national and acute health care facility level. Geneva: World Health Organization (2016). 27977095

[20] Methods In Medicine CAM. Retracted: the supervision and management mode of disinfection supply center improves the standardization of sterile goods Management in Clinical Departments. Comput Math Methods Med. (2023) 2023:9850961. doi: 10.1155/2023/9850961, PMID: 37416144 PMC10322391

[21] Lin S Shi Q Zhou N. Construction of a traceability module for food industry chain safety information based on internet of things technology. Front Public Health. (2022) 10:857039. doi: 10.3389/fpubh.2022.857039, PMID: 35712319 PMC9196936

[22] Jing W Mu Y Cai Y. Central sterile supply department (CSSD) management quality sensitive index constructed by management mode under the guidance of key point control theory and its effect on CSSD management quality: a retrospective study. Ann Palliat Med. (2022) 11:2050–60. doi: 10.21037/apm-22-594, PMID: 35817740

[23] Yang L Xun Q Xu J Hua D. Application of the defect management improvement mode under joint commission international standard to improve the instrument cleaning and disinfection effect and management quality in the central sterile supply department: a randomized trial. Ann Transl Med. (2022) 10:137. doi: 10.21037/atm-21-6610, PMID: 35284550 PMC8904978

[24] Wang L Cai X Cheng P. Application of a sub-specialties management model improves quality control in a central sterile supply department. BMC Health Serv Res. (2018) 18:385. doi: 10.1186/s12913-018-3214-7, PMID: 29843705 PMC5975497

[25] Gu Q Wang HF Fang Y Lu Y Shen Z Wang Y. Analysis of an improved workflow of endoscope reprocessing for bedside endoscopic diagnosis and treatment on COVID-19 patients. J Zhejiang Univ Sci B. (2020) 21:416–22. doi: 10.1631/jzus.B2000109, PMID: 32425010 PMC7210095

[26] Alfa MJ. Medical instrument reprocessing: current issues with cleaning and cleaning monitoring. Am J Infect Control. (2019) 47:A10–6. doi: 10.1016/j.ajic.2019.02.029, PMID: 31146843

[27] He W Qi C Ding L. The mechanism analysis on the farmers' motivation of using the quality traceability module based on TAM-ECM model. Sci Rep. (2023) 13:22283. doi: 10.1038/s41598-023-49795-7, PMID: 38097697 PMC10721799

[28] Lin K Chavalarias D Panahi M Yeh T Takimoto K Mizoguchi M. Mobile-based traceability module for sustainable food supply networks. Nat Food. (2020) 1:673–9. doi: 10.1038/s43016-020-00163-y, PMID: 37128031

[29] Troutner JC Harrell MV Seelen MT Daily BJ Levine WC. Using Real-Time Locating Systems to Optimize Endoscope Use at a Large Academic Medical Center. J Med Syst. (2020) 44:71. doi: 10.1007/s10916-020-1540-x 32078101

[30] Hu T Yi L Tang Y Chen Y Hu R. Enhancing nighttime surgical instrument cleaning efficiency: an ECRS-based approach. Med Sci Monit. (2023) 29:e940346. doi: 10.12659/MSM.940346, PMID: 37482678 PMC10375914

[31] Rutala WA Weber DJ. Disinfection and sterilization in health care facilities: an overview and current issues. Infect Dis Clin N Am. (2021) 35:575–607. doi: 10.1016/j.idc.2021.04.004, PMID: 34362535

[32] Zhang J Zhou H. Research on visualized traceability supervision module of medical equipment based on wireless local area network real-time positioning module. Zhongguo Yi Liao Qi Xie Za Zhi. (2021) 45:487–91. doi: 10.3969/j.issn.1671-7104.2021.05.004, PMID: 34628758

[33] Hu T Huang J Jiang S Hu R Huang Y Pan W. Improvement and implementation of central sterile supply department training program based on action research. BMC Nurs. (2024) 23:184. doi: 10.1186/s12912-024-01809-z, PMID: 38494483 PMC10946169

[34] Lesińska-Sawicka M. Using graphic medicine in teaching multicultural nursing: a quasi-experimental study. BMC Med Educ. (2023) 23:255. doi: 10.1186/s12909-023-04223-2, PMID: 37069640 PMC10111694

[35] Yang H Fan Y Chen Z Zhang S Wu H Hu X. Constructing a diversified online neurology teaching model under the COVID-19. Front Med (Lausanne). (2023) 9:1071414. doi: 10.3389/fmed.2022.1071414, PMID: 36698791 PMC9868292

[36] Nematian H Masoumnia AM Shakiba S Milan N Vahdati Z Oryadi Zanjani L. Comparison of instructor-led and video-based instruction in teaching suturing to medical students. J Surg Res. (2023) 287:134–41. doi: 10.1016/j.jss.2023.02.007, PMID: 36933544

[37] Stone R Cooke M Mitchell M. Undergraduate nursing students' use of video technology in developing confidence in clinical skills for practice: a moduleatic integrative literature review. Nurse Educ Today. (2020) 84:104230. doi: 10.1016/j.nedt.2019.104230, PMID: 31689584

[38] Forbes H Oprescu FI Downer T Phillips NM McTier L Lord B. Use of videos to support teaching and learning of clinical skills in nursing education: a review. Nurse Educ Today. (2016) 42:53–6. doi: 10.1016/j.nedt.2016.04.010, PMID: 27237353

[39] Basu D Bhattacharya S Mahajan A Ramanan VR Chandy M. Sterilization indicators in central sterile supply department: quality assurance and cost implications. Infect Control Hosp Epidemiol. (2015) 36:484–6. doi: 10.1017/ice.2014.40 25782908

[40] Whelan J. Current issues in reprocessing of medical and surgical instruments. Am J Infect Control. (2023) 51:1185–8. doi: 10.1016/j.ajic.2023.04.004, PMID: 37044263

[41] Seavey R. Using a moduleatic approach for adopting new technologies in sterile processing departments and operating rooms. Am J Infect Control. (2019) 47S:A67–71. doi: 10.1016/j.ajic.2019.01.016, PMID: 31146854

